# Supplementary material for: Halogen-Substituted Triazolethioacetamides as a Potent Skeleton for the Development of Metallo-β-Lactamase Inhibitors
Source: Molecules. 2019 Mar 25;24(6):1174. doi: 10.3390/molecules24061174 (PMC6471427; doi:10.3390/molecules24061174)
Supplement: Supplementary file 1 [file molecules-24-01174-s001.pdf]

Supporting Information

Halogen-substituted triazolethioacetamides as a potent skeleton for the development of metallo-β-lactamase inhibitors

Yi-Lin Zhang[1,*], Yong Yan[1], Lu-fan Liang[1], Jie Feng[1], Xue-jun Wang[1], Li Li[1], Ke-wu Yang[2]

Table of Contents

Synthetic Experimental Procedures 2

General 2

Synthesis and characterization 2

^{1}H and ^{13}C NMR data of halogen-substituted triazolethioacetamides 2

1. N-(2-chlorophenyl)-5-(5-phenyl-4H-1,2,4-triazolyl)thioacetamide 2
2. N-(3-chlorophenyl)-5-(5-phenyl-4H-1,2,4-triazolyl)thioacetamide 2
3. N-(4-chlorophenyl)-5-(5-phenyl-4H-1,2,4-triazolyl)thioacetamide 3
4. N-(3,4-dichlorophenyl)-5-(5-phenyl-4H-1,2,4-triazolyl)thioacetamide 3
5. N-(2,4-dichlorophenyl)-5-(5-phenyl-4H-1,2,4-triazolyl)thioacetamide 3
6. N-(4-chloro-2-nitrophenyl)-5-(5-phenyl-4H-1,2,4-triazolyl)thioacetamide 3
7. N-(5-chloro-2-nitrophenyl)-5-(5-phenyl-4H-1,2,4-triazolyl)thioacetamide 3
8. N-(2-chloro-4-nitrophenyl)-5-(5-phenyl-4H-1,2,4-triazolyl)thioacetamide 4
9. N-(2-fluorophenyl)-5-(5-phenyl-4H-1,2,4-triazolyl)thioacetamide 4
10. N-(3-fluorophenyl)-5-(5-phenyl-4H-1,2,4-triazolyl)thioacetamide 4
11. N-(4-fluorophenyl)-5-(5-phenyl-4H-1,2,4-triazolyl)thioacetamide 4
12. N-(2,6-difluorophenyl)-5-(5-phenyl-4H-1,2,4-triazolyl)thioacetamide 4
13. N-(4-(trifluoromethoxy)phenyl)-5-(5-phenyl-4H-1,2,4-triazolyl)thioacetamide 5

Synthetic Experimental Procedures

General

General chemicals were purchased from TCI and were used without further purification. All antibiotics used were purchased from Sigma-Aldrich.^{1}H NMR and ^{13}C NMR spectra were recorded on a NMR spectra were recorded with a Bruker DRX 600 MHz spectrometer. The peaks patterns are indicated as follows: s, singlet; d, doublet; t, triplet; q, quartet; dd, doublet doublet; m, multiplet. The spectra were recorded with TMS as internal standard. Coupling constants (J) were reported in hertz (Hz). Chemical shifts were given in part per million (ppm) on the delta scale. Analytical Thin Layer Chromatography (TLC) was carried out on silica gel F_{254} plates with visualization by ultraviolet radiation. HRMS spectra were recorded on a Bruker MicrOTOF-Q II mass spectrometer. Inhibition studies were performed on an Agilent-8453 UV-visible spectrometer.

Synthesis and characterization

N-substituted-2-chloroacetamides (**N1-13**) were first prepared by acylation between substituted anilines and chloroacetyl chloride. The Methyl 2-hydroxybenzoate (**s1**) were prepared by esterifying of salicylic acid and converted into hydrazides **s2** by condensing with hydrazine. The hydrazides reacted with NH_4SCN under basic condition to give aroylthiourea **s3**. The thiourea was stirred with NaOH under reflux to afford 2-(5-mercapto-4H-1,2,4-triazol-3-yl)phenol (**s4**).

Halogen-substituted triazolethioacetamides (1-13):

A solution of 2-(5-mercapto-4H-1,2,4-triazol-3-yl)phenol (**s4**) (3 mmol) and NaOH (3.6 mmol) dissolved in H_2O (15 mL) was added in a 50 mL three-neck round bottomed flask, kept stirring for 30 min. After N-substituted-2-chloroacetamides (**N1-13**) (3 mmol) dissolved in hot ethanol (5 mL) was added drop wise, the reaction mixture was heated to reflux for 6 h. Reaction mixture was cooled to RT and neutralized with 5 M HCl to a pH approximately 7.0. The resulting white solid was collected by filtration, washed with H_2O repeatedly (3×80 mL) and dried in vacuo to obtain **1-13**.

^{1}H and ^{13}C NMR data of halogen-substituted triazolethioacetamides:

1. N-(2-chlorophenyl)-5-(5-phenyl-4H-1,2,4-triazolyl)thioacetamide (1)

White powder, yield 66.2 %, ^{1}H NMR (600 MHz, DMSO-d_6): δ 7.87 (s, 2H), 7.55 (s, 2H), 7.43 (s, 1H), 7.19 (s, 2H), 6.83 (s, 2H), 4.12 (s, 2H); HRMS $[M-H]^-$ (*m/z*) for $C_{16}H_{13}ClN_4O_2S$: calcd. 360.0448, obsd. 359.0448.

2. N-(3-chlorophenyl)-5-(5-phenyl-4H-1,2,4-triazolyl)thioacetamide (2)

White powder, yield 62.5 %,^{1}H NMR (600 MHz, DMSO-d_6): δ 10.52 (s, 1H), 7.89 (d, J = 7.8 Hz, 1H), 7.80 (s, 1H), 7.45 (d, J = 8.2 Hz, 1H), 7.37-7.34 (m, 1H), 7.34-7.32 (m, 1H), 6.99 (d, J = 8.2 Hz, 1H), 6.97-6.89 (m, 2H), 4.12 (s, 2H). ^{13}C NMR (151 MHz, DMSO-d_6): δ 166.78, 165.98, 155.70, 152.94, 140.44, 133.21, 133.20, 130.59, 127.47, 123.24, 119.53, 118.69, 117.62, 116.73, 113.75, 36.33.HRMS $[M-H]^-$ (m/z) for $C_{16}H_{13}ClN_4O_2S$: calcd. 360.0448, obsd. 359.0446.

3. N-(4-chlorophenyl)-5-(5-phenyl-4H-1,2,4-triazolyl)thioacetamide (3)

White powder, yield 71.4 %, ^{1}H NMR (600 MHz, DMSO-d_6): δ 10.48 (s, 1H), 7.89 (d, J = 7.8 Hz, 1H), 7.62 (d, J = 8.8 Hz, 2H), 7.35 (t, J = 12.2 Hz, 2H), 7.32 (t, J = 7.0 Hz, 1H), 6.99 (d, J = 8.2 Hz, 1H), 6.93 (t, J = 7.9 Hz, 1H), 4.12 (s, 2H). ^{13}C NMR (151 MHz, DMSO-d_6): δ 166.50, 155.72, 137.98, 131.68, 128.89, 128.78, 127.41, 127.09, 127.05, 120.77, 119.53, 116.75, 112.55, 36.35. HRMS $[M-H]^-$ (m/z) for $C_{16}H_{13}ClN_4O_2S$: calcd. 360.0448, obsd. 359.0447.

4. N-(3,4-dichlorophenyl)-5-(5-phenyl-4H-1,2,4-triazolyl)thioacetamide (4)

Yellow powder, yield 86.1 %, ^{1}H NMR (600 MHz, DMSO-d_6):δ10.65 (s, 1H), 7.98 (d, J = 2.3 Hz, 1H), 7.88 (d, J = 7.8 Hz, 1H), 7.57 (d, J = 8.2 Hz, 1H), 7.49 (dd, J = 8.8, 2.4 Hz, 1H), 7.32 (t, J = 7.7 Hz, 1H), 6.99 (d, J = 8.2 Hz, 1H), 6.93 (t, J = 7.5 Hz, 1H), 4.12 (s, 2H). ^{13}C NMR (151 MHz, DMSO-d_6): δ166.97, 155.71, 139.08, 131.67, 131.14, 130.82, 127.43, 124.97, 120.42, 119.51, 119.28, 116.73, 112.58, 36.32.HRMS $[M-H]^-$ (m/z) for $C_{16}H_{12}Cl_2N_4O_2S$: calcd. 394.0058, obsd. 393.0070.

5. N-(2,4-dichlorophenyl)-5-(5-phenyl-4H-1,2,4-triazolyl)thioacetamide (5)

Yellow powder, yield 65.6 %, ^{1}H NMR (600 MHz, DMSO-d_6): δ 9.89 (s, 1H), 7.91 (d, J = 7.8 Hz, 1H), 7.87 (d, J = 8.8 Hz, 1H), 7.64 (s, 1H), 7.41 (dd, J = 8.8, 2.1 Hz, 1H), 7.33 (t, J = 7.7 Hz, 1H), 7.00 (d, J = 8.2 Hz, 1H), 6.94 (t, J = 7.4 Hz, 1H), 4.18 (s, 2H). ^{13}C NMR (151 MHz, DMSO-d_6): δ 167.18, 155.69, 133.98, 131.79, 129.24, 128.98, 127.72, 127.63, 126.42, 125.99, 119.53, 116.72, 35.78. HRMS $[M-H]^-$ (m/z) for $C_{16}H_{12}Cl_2N_4O_2S$: calcd. 394.0058, obsd. 393.0071.

6. N-(4-chloro-2-nitrophenyl)-5-(5-phenyl-4H-1,2,4-triazolyl)thioacetamide (6)

Brown powder, yield 63.5 %, ^{1}H NMR (600 MHz, DMSO-d_6): δ 10.89 (s, 1H), 8.10 (s, 1H), 8.07 (d, J = 8.8 Hz, 1H), 7.89 (dd, J = 7.8, 1.6 Hz, 1H), 7.42 (d, J = 8.8 Hz, 1H), 7.32 (t, J = 7.8 Hz, 1H), 6.98 (d, J = 7.7 Hz, 1H), 6.93 (t, J = 7.6 Hz, 1H), 4.17 (s, 2H). ^{13}C NMR (151 MHz, DMSO-d_6): δ 167.53, 155.67, 154.72, 138.98, 133.29, 131.77, 127.55, 127.30, 124.79, 123.30, 119.54, 117.74, 116.71, 115.63, 112.48, 36.10. HRMS [M-H]- (m/z) for $C_{16}H_{12}ClN_5O_4S$: calcd. 405.0299, obsd. 404.0305.

7. N-(5-chloro-2-nitrophenyl)-5-(5-phenyl-4H-1,2,4-triazolyl)thioacetamide (7)

Brown powder, yield 71.4 %, ^{1}H NMR (600 MHz, DMSO-d$_6$): δ 10.80 (s, 1H), 8.08 (s, 1H), 7.89 (d, J = 6.6 Hz, 2H), 7.80 (d, J = 8.8 Hz, 1H), 7.32 (t, J = 7.8 Hz, 1H), 6.99 (d, J = 8.2 Hz, 1H), 6.94 (t, J = 7.5 Hz, 1H), 4.16 (s, 2H). ^{13}C NMR (151 MHz, DMSO-d$_6$): δ 198.52, 187.05, 172.94, 165.59, 163.14, 161.91, 160.02, 158.92, 157.59, 156.27, 150.93, 148.09, 143.85, 35.89. HRMS [M-H]$^-$ (m/z) for $C_{16}H_{12}ClN_5O_4S$: calcd. 405.0299, obsd. 404.0304.

8. N-(2-chloro-4-nitrophenyl)-5-(5-phenyl-4H-1,2,4-triazolyl)thioacetamide (8)

Brown powder, yield 87.3 %,^{1}H NMR (600 MHz, DMSO-d$_6$): δ 10.15 (s, 1H), 8.83 (d, J = 2.5 Hz, 1H), 7.99 (dd, J = 8.8, 2.6 Hz, 1H), 7.90 (dd, J = 7.8, 1.5 Hz, 1H), 7.79 (d, J = 8.8 Hz, 1H), 7.33 (t, J = 7.8 Hz, 1H), 6.99 (d, J = 8.2 Hz, 1H), 6.93 (t, J = 7.5 Hz, 1H), , 4.25 (s, 2H). ^{13}C NMR (151 MHz, DMSO-d$_6$): δ 167.89, 155.68, 146.39, 135.84, 131.82, 131.35, 130.79, 127.69, 120.24, 119.54, 118.31, 116.72, 116.67, 35.89. HRMS [M-H]$^-$ (m/z) for $C_{16}H_{12}ClN_5O_4S$: calcd. 405.0299, obsd. 404.0320.

9. N-(2-fluorophenyl)-5-(5-phenyl-4H-1,2,4-triazolyl)thioacetamide (9)

White powder, yield 56.9 %, ^{1}H NMR (600 MHz, DMSO-d$_6$): δ 10.10 (s, 1H), 7.92 (s, 2H), 7.32 (t, J = 7.8 Hz, 1H), 7.27-7.22 (m, 1H), 7.16-7.14 (m, 2H), 7.01 (s, 1H), 6.94 (t, J = 7.5 Hz, 1H), 4.19 (s, 2H). ^{13}C NMR (151 MHz, DMSO-d$_6$): δ 166.91, 155.76, 131.69, 127.50, 126.13, 126.05, 125.40, 125.36, 124.46, 124.44, 123.76, 119.50, 116.75, 115.61, 115.48, 35.89. HRMS [M-H]$^-$ (m/z) for $C_{16}H_{13}FN_4O_2S$: calcd. 344.0743, obsd. 343.0735.

10. N-(3-fluorophenyl)-5-(5-phenyl-4H-1,2,4-triazolyl)thioacetamide (10)

White powder, yield 65.5 %, ^{1}H NMR (600 MHz, DMSO-d$_6$): δ 10.54 (s, 1H), 7.89 (dd, J = 7.8, 1.6 Hz, 1H), 7.58 (dt, J = 11.7, 2.1 Hz, 1H), 7.37-7.34 (m, 1H), 7.34-7.32 (m, 1H), 7.31-7.29 (m, 1H), 6.99 (dd, J = 8.2, 0.8 Hz, 1H), 6.95-6.91 (m, 1H), 6.91-6.86 (m, 1H), 4.12(s, 2H). ^{13}C NMR (151 MHz, DMSO-d$_6$): δ 166.73, 163.02, 161.42, 155.70, 140.72, 131.72, 130.53, 127.47, 119.53, 116.74, 114.97, 114.95, 112.52, 109.98, 106.01, 36.35. HRMS [M-H]$^-$ (m/z) for $C_{16}H_{13}FN_4O_2S$: calcd. 344.0743, obsd. 343.0755.

11. N-(4-fluorophenyl)-5-(5-phenyl-4H-1,2,4-triazolyl)thioacetamide (11)

White powder, yield 56.9%, ^{1}H NMR (600 MHz, DMSO-d$_6$): δ 10.57 (s, 1H), 7.94 (s, 1H), 7.64 (s, 2H), 7.31 (t, J = 7.5 Hz, 1H), 7.14 (t, J = 8.0 Hz, 2H), 7.02 (d, J = 8.0 Hz, 1H), 6.93 (t, J = 7.2 Hz, 1H), 4.13 (s, 2H). ^{13}C NMR (151 MHz, DMSO-d$_6$): δ 166.30, 158.97, 157.38, 155.82, 135.51, 135.49, 131.67, 127.47, 121.03, 119.52, 116.80, 115.42, 112.56, 36.30. HRMS [M-H]$^-$ (m/z) for $C_{16}H_{13}FN_4O_2S$: calcd. 344.0743, obsd. 343.0729.

12. N-(2,6-difluorophenyl)-5-(5-phenyl-4H-1,2,4-triazolyl)thioacetamide (12)

White powder, yield 62.3%,^{1}H NMR (600 MHz, DMSO-d_6): δ 10.08 (s, 1H), 7.93 (d, J = 7.8 Hz, 1H), 7.33 (m, 2H), 7.14 (t, J = 8.2 Hz, 2H), 7.00 (d, J = 8.2 Hz, 1H), 6.97-6.91 (m, 1H), 4.18 (s, 2H). ^{13}C NMR (151 MHz, DMSO-d_6): δ 166.67, 158.54, 156.89, 155.75, 131.72, 128.16, 127.50, 119.54, 116.75, 114.35, 112.55, 112.01, 111.88, 35.12. HRMS $[M-H]^-$ (m/z) for $C_{16}H_{12}F_2N_4O_2S$: calcd. 362.0649, obsd. 361.0648.

13. N-(4-(trifluoromethoxy)phenyl)-5-(5-phenyl-4H-1,2,4-triazolyl)thioacetamide (13)

White powder, yield 86.4%, ^{1}H NMR (600 MHz, DMSO-d_6):δ 10.52 (s, 1H), 7.89 (d, J = 7.8 Hz, 1H), 7.70 (d, J = 8.9 Hz, 1H), 7.32 (m, 3H), 6.99 (d, J = 8.2 Hz, 1H), 6.93 (t, J = 7.5 Hz, 1H), 4.13 (s, 2H). ^{13}C NMR (151 MHz, DMSO-d_6): δ 166.58, 155.71, 143.75, 138.23, 131.75, 127.46, 122.76, 121.80, 121.07, 120.59, 119.55, 119.38, 116.75, 112.51, 36.30. HRMS $[M-H]^-$ (m/z) for $C_{17}H_{13}F_3N_4O_3S$: calcd. 410.0660, obsd. 409.0674.
